# Quality of life and associated factors in Swiss trans people: a cross-sectional study

**DOI:** 10.3389/fpsyt.2023.1233625

**Published:** 2024-01-04

**Authors:** David Garcia Nuñez, Giulia Frigerio, Laura D. Perler, Tiziana Jäggi, Verena Schönbucher, Roland von Känel

**Affiliations:** ^1^Center for Gender Variance, University Hospital Basel, Basel, Switzerland; ^2^Department of Consultation-Liaison Psychiatry and Psychosomatic Medicine, University Hospital Zurich, University of Zurich, Culmannstrasse, Zürich, Switzerland; ^3^Department of General Surgery, University Hospital Geneva, Rue Gabrielle-Perret-Gentil, Geneva, Switzerland; ^4^Department of Psychology, University of Fribourg, Fribourg, Switzerland; ^5^Praxis für Psycho- und Sexualtherapie, Zürich, Switzerland

**Keywords:** gender dysphoria, gender incongruence, LGBT, mental health, discrimination, minority stress, quality of life

## Abstract

**Background:**

Experiences of stressful life events during transition may have a negative impact on quality of life (QoL) in trans persons. Little attention has been paid to this population in Switzerland, resulting in sparse data on their QoL and associated social factors.

**Methods:**

30 participants were recruited during their medical transition treatment and surveyed on their experiences within this time period (13 months after the first medical intervention on average). After performing a diagnostic interview to evaluate their mental health, health-related QoL, psychological distress, self-esteem and the impact of life events that occurred in the last six months on participants were further assessed.

**Results:**

Approximately 17% of participants had suffered from major depression, 43% reported having had suicidal thoughts or having attempted suicide, and 43% suffered from an anxiety disorder. Psychological distress was twice as high compared to the norm values of the cis population. With regard to QoL, trans individuals showed impairments in the mental domain. Stressful life events were particularly evident on a psychological and social level. Analysis showed a negative correlation between impact of life events and mental QoL and between psychological distress and mental QoL. At the same time, there was a positive correlation between self-esteem and mental QoL. Psychological distress and self-esteem emerged as independent significant predictors of mental QoL.

**Conclusion:**

This study shows lowered mental QoL and associations of low mental QoL with psychological distress, low self-esteem and stressful life events in trans individuals in Switzerland. The findings concur with the Gender Minority Stress Model and point out that medical transition must not be viewed in isolation but must be embedded in the framework of integrative psychosocial support.

## Background

Trans persons are subject to stigmatization (discrimination, stereotyped expectations, rejection experiences) (1), which are based on socially embodied cis heteronormative assumptions (2, 3). According to a comparative European analysis, more than half of interviewed trans persons (55%) had been discriminated in the previous year because of their gender identity and 17% had experienced physical and/or sexual attack (4). Switzerland is no exception to this. The latest Swiss LGBTIQ+ Panel reported that surveyed trans people experienced discrimination in the past 12 months via structural discrimination (81%), jokes (80%) and physical violence (14.5%) (5). Furthermore, one fourth (25%) of interviewed trans people have not come out to friends at all and less than half (44%) have come out to most/all family member (5). In the same line, a national report showed a 20% rate of unemployment among trans people. This is highly increased compared to the cis^1^ population (2–3%) and can be attributed to the risk of losing one’s job at the start of social transition (6).

Members of a stigmatized minority are at higher risk for poor health outcomes, including depression, anxiety, substance use, and suicidality (7, 8). In the trans population, this can be seen in terms of poor mental health (3, 8) and low quality of life (QoL) (9). Specifically, meta-analysis data suggest that mental health-related QoL of trans people is significantly poorer compared to the general population, with a medium to large effect size (standard mean difference = 0.78) (10). Studies identified several factors which influence the QoL of trans populations in a positive way: the non-existence of psychopathological symptoms (11), the performance of a medical transition (12–14), and social support (15). However, not all studies could replicate these findings. For example, in a survey conducted with 143 Swiss trans people after medical transition, only preliminary associations between mental health-related QoL and completion of medical transition measures were established (16). The categories “gender” (male/ female/ non-binary) and “work situation” (as a proxy for status loss) emerged as significant factors. These results are consistent with findings from other studies highlighting effects of social factors (e.g., low income, unemployment, family support) on the QoL in trans persons (15, 17–19). With one exception, these studies did not explicitly investigate how social factors are linked to the stigmatized situation of the trans population in general, which is a significant research gap (19).

Estimates of prevalence of gender dysphoria ranges between 0.1–1.1% and similar values are expected for Switzerland (20). In 2022, the Swiss Federal Statistical Office counted 525 hospitalizations related to gender-affirming interventions concerning 486 trans individuals. Based on these numbers, clinicians in Switzerland expect that about 600 people start medical transition each year in Switzerland (21). However, these numbers should be taken with caution as not all trans individuals transition medically and an unknown portion underwent surgery abroad (22). In Switzerland, health care insurance is mandatory and most medical gender-affirming interventions carried out in Switzerland are covered by the insurances. The ICD-10 diagnosis of “transsexualism” (ICD-10: F64.0) is needed for access to medical measures.

To understand the QoL of this population in a more comprehensive way, the Gender Minority Stress model (GMSM) aims to explain mechanisms on how stigmatizing experiences affect the mental and physical health of the trans population (2, 23). Further, the GMSM does not only postulate direct and indirect ways in which stigmatizing processes can negatively affect the individual trans person. It also explains how certain protective factors such as self-esteem can develop a buffering effect on various negative bio-psycho-social outcomes (24). Systematic reviews and meta-analyses have provided first indications for the plausibility of the GMSM (25, 26).

In line with demands to understand the situation of trans people not only from an isolated medical or even psychiatric perspective, there is a need for studies that examine both QoL and the psycho-social situation concurrently in order to determine relevant relationships between these domains (16). Previous studies did not measure the correlation between stressful life events, intrapsychic resources and QoL using the framework of the GMSM. From a clinical standpoint, this omission is problematic as it fails to consider crucial sources of psychological suffering within the transgender population. Such information could ultimately improve understanding of trans people’s needs and provide physicians and psychotherapists with guidance on intervention options to support trans people.

### Objectives

The first purpose of this study was to assess the QoL of Swiss trans people during their transition with the hypothesis that participants will have a lower QoL compared to the general population (aim 1). Simultaneously, the study records participants’ mental health status, psychological stress level and stressful situations they have experienced as members of a gender minority. We assume that, on average, they will report multiple mental health problems, numerous experiences of discrimination and few intrapersonal protection mechanisms (aim 2). Finally, the study analyzes the associations between QoL, mental health problems, psychological stress level, external stressors and intrapsychic ressources. Based on the GMSM, it can be assumed that both external experiences of discrimination and a lack of intrapsychic protection mechanisms will have a negative impact on the QoL of the respondents (aim 3).

## Methods

### Participants

Participants (*N* = 30) were recruited through the Department of Psychiatry and Psychotherapy of the University Hospital in Zurich, trans support groups, and informal peer networks. Participants were included if they had a diagnosis of Gender Dysphoria (GD) and were at least 18 years old. People with no knowledge of German were excluded from the study. Trans persons who were recruited by the psychiatric team (*N* = 18) needed no further diagnostic confirmation of the GD status. For the other 12 participants, a preliminary clinical interview confirmed the GD diagnosis. All participants showing symptoms of an acute psychotic episode or who had acute suicidal ideation were excluded from the study.

### Data collection

Participants were screened as part of a larger project to study the psychological consequences of gender minority stress experienced by trans persons. After signing for informed consent, the participants were examined by means of a structured diagnostic interview. Subsequently, they completed a pencil and paper questionnaire. The interviews and survey were carried out between July and December 2013.

### Instruments

#### Aim 1: QoL [short form (36) health survey (SF-36)]

To measure QoL the German version of the SF-36 was used (27). This self-assessment tool evaluates the health-related QoL in eight domains (vitality, physical functioning, bodily pain, general health perceptions, physical role limitations, emotional role limitations, social functioning, and mental health). The Physical Component Summary (PCS) and Mental Component Summary (MCS) are combinations of the eight scale scores listed above (28). For each subscale the scores range from 0 (worst possible health state) to 100 (best possible health state). Reliability alpha coefficients ranged from a low of α = 0.65 to a high of α = 0.94 across the different scales (Mdn = 0.85) in our sample.

#### Aim 2: Mental health diagnoses [Mini international neuropsychiatric interview plus (MINI-PLUS)]

The MINI-PLUS is a structured diagnostic interview (29), developed to facilitate the diagnosis of psychiatric patients according to DSM-IV (30) and ICD-10 (31) criteria in both clinical practice and research. It is organized into independent sections to optimize its sensitivity as a diagnostic tool. The MINI 6.0 comprises modules for 17 psychiatric diagnoses according to ICD-10. Questions are phrased to allow only “yes” or “no” answers and examples are provided to facilitate responses.

#### Aim 2: Psychological stress level [symptom checklist 9-K (SCL-K-9)]

Subjectively perceived overall psychological distress was measured with the German adaptation of the short version of the Symptom Checklist 90-R (SCL-K-9) (32). It consists of 9 items and measures only a general severity factor, where high values correspond to higher psychological distress. This instrument had a satisfactory Cronbach’s alpha (α = 0.87).

#### Aim 2: Externally induced stressors [social readjustment rating scale (SRRS)]

The Social Readjustment Rating Scale is a well-established instrument for quantifying the impact of stressful life events and measures the occurrence of 43 different socially burdening situations during the last six months (33). It assesses trans specific life events in addition to general ones. The German version of this questionnaire was used in this study to measure externally induced stressors (stressful life events). The higher the sum, the more stressful events a participant has experienced. In the absence of an instrument validated in German to record minority stress levels, this instrument was selected due to its excellent reliability in both healthy adults (r = 0.96–0.89) and patients (r = 0.91 to 0.70). The level of stress is classified as low (0–149), mild (150–199), moderate (200–299), or major (>300) according to the total scores of added items.

#### Aim 2: Intrapersonal protective factor [Rosenberg self esteem scale (RSES)]

Self-esteem was determined with the German version of the RSES, a widely used instrument to measure a person’s global self-esteem with 10 questions to be answered on a four-point scale with responses ranging from strongly disagree (1) to strongly agree (4) (34). The scale assesses the degree to which respondents are satisfied with their lives as well as how good or bad participants feel about themselves. A lower score reflects good self-esteem. High reliabilities (Cronbach’s alphas) have been reported for various samples that range from α = 0.77 to α = 0.88 (35).

#### Sociodemographic questionnaire

During the assessment, important sociodemographic and transition-related variables were collected. These included first experience of gender incongruence, first acceptance of their gender incongruence, changing of name and/or pronouns, start of hormonal therapy, and time since surgical interventions.

### Data analysis and statistics

After performing a statistical analysis of the descriptive data (aim 1 and 2), bivariate correlations and regression analysis were used to analyze the association between each of the independent variables (impact of live events, self-esteem, and psychological distress) and the dependent variable (QoL) (aim 3). To identify statistically significant differences, a value of *p* <0.05 was employed. All statistical analyses were performed using R Version 4.1.

## Results

### Descriptive statistics

#### Sociodemographic data

Regarding gender identities, 14 participants referred to their gender as “female,” 11 as “male” and five as “other” (four participants were assigned female at birth, one participant was assigned male at birth) (see additional data). We will refer to participants as “trans feminine” and “trans masculine,” instead of “trans women” and “trans men,” respectively. This partition groups them better in terms of their undertaken medical interventions. In the final sample, the mean age of the 15 trans feminine and 15 trans masculine individuals was 34.6 ± 12.6 years. There was no significant difference between the two groups except for the circumstance that trans feminine subjects tend to live alone more often (*p* = 0.020). For all the sociodemographic categories, no significant difference was found between participants recruited by the psychiatric team and those recruited outside the clinic.

#### State of transition

Eight trans persons (four feminine/ four masculine) had exclusively undertaken social transition measures at the time of the study (Table 1). Nine trans feminine and six trans masculine persons were taking hormone therapy but had not performed surgical interventions yet. Finally, seven participants (two feminine/ five masculine) also had undergone surgeries. There were no statistically significant differences between the transition groups, as well as between participants recruited by the psychiatric team and those recruited outside the clinic. However, trans feminine persons (four individuals) were taking significantly more antidepressants than trans masculine persons (one individual).

**Table 1:** State of transition.

	Trans population M ± SD	Trans feminine M ± SD	Trans masculine M ± SD	*p*
Social transition (age in years)				
First Experience	6.81 ± 3.48	7.47 ± 3.70	6.00 ± 3.13	0.301
First Acceptance	19.79 ± 12.75	22.27 ± 14.86	16.92 ± 9.58	0.447
Outing	29.04 ± 12.13	32.20 ± 13.81	25.38 ± 9.04	0.240
Medical Transition (months)				
Hormone Therapy	22.45 ± 16.13	19.49 ± 13.06	25.72 ± 19.14	0.557
SRS	36.37 ± 45.94	67.70 ± 90.58	23.85 ± 20.67	0.063
Additional Measures (N)	14	10	4	0.067

SRS: Sex Reassignment Surgery. The analysis of all variables was conducted with chi-square for qualitative data and Wilcoxon test for quantitative data (trans feminine vs. trans masculine).

#### Aim 1: Quality of life

Health-related quality of life (QoL) is a broad concept that focuses on physical, psychological and social aspects of the life conditions of a specific patient group. In this study, four somatic (physical functioning, physical role limitations, bodily pain, general health perceptions) and four mental (vitality, social functioning, emotional role functioning, and mental well-being) components of QoL are surveyed (Table 2). No statistically significant difference was found between the two gender groups concerning the QoL physical as well as mental health-related component summary scores of the SF-36. Similar results were found when comparing participants recruited outside the clinic to those recruited by the psychiatric team. Bullinger and Kirchberger (36) have assessed SF-36 values for a German speaking norm population. In comparison with the reported scores by trans individuals in this study, both standard values for physical component summary scores (PCS) in the feminine and masculine norm population were lower (feminine: M ± SD = 49.09 ± 10.6; masculine: M ± SD = 51.42 ± 9.62) compared to those of trans persons. This ratio was reversed for the mental component summary scores (MCS). Both the feminine (M ± SD = 50.71 ± 8.39) and the masculine (M ± SD = 52.44 ± 7.7) norm population showed higher values than the participants of the respective gender.

**Table 2:** Quality of life.

	Trans population M±SD	Trans feminine M±SD	Trans masculine M±SD	*p*
Physical Component Summary (PCS)	54.40±6.39	54.07±6.08	54.74±6.88	0.903
Physical Functioning	93.83±9.80	93.67±9.54	94.00±10.39	0.894
Physical Role Limitations	85.83±25.16	83.33±27.82	88.33±22.89	0.663
Bodily Pain	82.77±19.33	83.73±20.42	81.80±18.83	0.741
General Health Perceptions	77.43±19.37	75.00±19.89	79.87±19.21	0.368
Mental Component Summary (MCS)	46.89±10.72	46.77±11.00	47.01±10.81	0.903
Vitality	55.83±19.21	55.67±21.20	56.00±17.75	0.967
Social Functioning	86.67±18.55	87.50±21.13	85.83±16.28	0.490
Emotional Role Limitations	78.89±32.14	80.00±30.34	77.78±34.88	0.962
Mental Health	70.67±18.77	69.07.56±20.92	72.27±16.93	0.692

The analysis of all variables was conducted with Wilcoxon test (trans feminine vs trans masculine).

#### Aim 2: Mental health

No significant gender-specific differences were found concerning psychiatric diagnosis, even considering the two different recruitment patterns (Table 3). Out of five (16.67%) participants who had a major depression, four of them were trans feminine. In total, six trans feminine and seven trans masculine persons (43.33%) reported suicidal thoughts or had attempted suicide during their lifetime. Further, the highest prevalence was found for anxiety disorders (agoraphobia: 16%, social phobia 10%, generalized anxiety disorder 16%). The gender ratio for anxiety disorders was unbalanced with a higher percentage of trans feminine individuals (60%) affected compared to trans masculine individuals (40%). In total, 20 (66.67%) trans individuals had at least one psychiatric diagnosis, 17 (56.67%) at least two and 12 (40%) at least three. Regarding comorbidities, no significant gender difference was observed.

**Table 3:** Psychiatric diagnoses.

	*n* (%)	Trans feminine	Trans masculine
Affective Disorders			
Previous Depression	1 (3.33%)	1	0
Melancholic Features	1 (3.33%)	1	0
Dysthymia	3 (10%)	1	2
Obsessive-Compulsive Disorder	0 (0%)	0	0
Anxiety Disorders			
Agoraphobia	5 (16.67%)	3	2
Social Phobia	3 (10%)	2	1
Generalized Anxiety Disorder	5 (16.67%)	3	2
Post-Traumatic Stress Disorder	0 (0%)	0	0
Psychoactive Substance Use Disorders			
Alcohol Abuse Disorder	0 (0%)	0	0
Alcohol Misuse Disorder	3 (10%)	1	2
Substance Abuse Disorder	1 (3.33%)	1	0
Earlier Psychotic Episode	1 (3.33%)	1	0
Somatoform Disorders			
Somatization Disorder	2 (6.67%)	0	2
Pain Disorder	2 (6.67%)	1	1
Attention Deficit Disorder	2 (6.67%)	0	2
Eating Disorders			
Anorexia nervosa	0 (0%)	0	0
Bulimia nervosa	0 (0%)	0	0

#### Aim 3: Psychological distress

Participants’ values for the SCL-K-9 ranged from 0 to 22 (trans feminine: M ± SD = 8.20 ± 6.97; trans masculine M ± SD = 8.93 ± 6.95), without any statistically significant difference between genders (Wilcoxon test, *p* = 0.724). The values of the trans population were twice as high compared to the norm values of the cis population (cis feminine: M ± SD = 3.91 ± 4.95; cis masculine: M ± SD = 3.28 ± 4.53) (37).

#### Aim 2: Stressful life events

Life events burdened subjects in a different manner, with values ranging from 0 to 643, with participants already in therapy at the University Hospital Zurich having a slightly higher but not significantly different mean of burdening events (moderate vs. mild life stress: 203 vs. 150; *p* = 0.267) (see additional data). The Wilcoxon test showed also no statistically significant difference between trans feminine and trans masculine subjects for the sum of the SRRS (*p* = 0.901). In the last six months (i.e., during the timespan participants have been in social or/and medical transition), about 33% of participants had been subjected to “major business readjustment” and 30% had experienced a “major financial change.” Simultaneously, 11 people had had a “major change in usual type and/or amount of recreation,” while nine had had a change in personal habits such as quitting smoking. Concerning stress-related behaviors, 30% had gone through a modification of eating habits and 40% of sleeping habits. Approximately 43% reported a change in living conditions (i.e., a lot more or less food intake) and 30% changed residence.

#### Aim 2: Self-esteem

The maximum score in the RSES is 40, with a range from 11 to 30 in our participants and a mean value of 25.1 for trans feminine and 22.4 for trans masculine subjects and no significant difference between individuals; this was independent of whether participants were already followed by the psychiatric team or not. However, these numbers are much higher compared to those of the general population (M ± SD = 4.92 ± 0.82) found in literature (38). This suggests a much lower self-esteem in the trans population.

#### Aim 3: Bivariate analyses

Correlations between QoL (SF-36 and its subscales) and the dependent variables are listed in Table 4. Correlation analysis indicated that the impact of stressful life events (SRRS) has a significantly weak negative correlation with mental health subscales (*p* < 0.05). Further, besides physical functioning, all subscales of SF-36 were significant negatively associated with psychological distress (SCL-K-9). Significant positive correlations were reported between the mental subscales of SF-36 and self-esteem (RSES). Analyses showed a negative correlation between stress impact (SRRS) and mental health-related QoL (MCS) respectively between psychological distress (SCL-K-9) scores and MCS (Table 4). Conversely, a positive correlation between self-esteem (RSES) and MCS was found (note the reverted scale). No significant correlations were found for physical health-related QoL (PCS).

**Table 4:** Bivariate Correlations of the SF-36 and its Subscales (QoL) and related factors.

SF-36	PF	RP	BP	GH	VT	SF	RE	MH	PCS	MCS
SRRS	−0.15	−0.06	−0.56 ^**^	−0.23	−0.33	−0.36	−0.28	−0.37 *	0.15	−0.48 ^**^
SCL-K-9	−0.35	−0.49 ^**^	−0.57 ^**^	−0.52 ^**^	−0.80 ^***^	−0.78 ^***^	−0.72 ^***^	−0.83 ^***^	−0.21	−0.82 ^***^
RSES	0.20	0.42 ^*^	0.16	0.38 ^*^	0.58 ^***^	0.50 ^**^	0.67 ^***^	0.60 ^***^	−0.06	0.67 ^***^

The analysis of all variables was conducted using Spearman’s correlation. SF-36: Short Form 36; PF = physical functioning, RP = role physical, BP = bodily pain, GH = general health, VT = vitality, SF = social functioning, RE = role emotional, MH = mental health, PCS: Physical Component Score, MCS: Mental Component Score; SCL-K-9: Symptom Checklist short version-9, SRRS: Social Readjustment Rating Scale2, RSES: Rosenberg Self Esteem Scale.

* indicates *p* < 0.1. ** indicates *p* < 0.01. *** indicates *p* < 0.001.

#### Aim 3: Regression analyses

Based on the outcomes of the bivariate analyses, the influence of the independent variables on mental health-related QoL (MCS) was assessed. Table 5 presents the results of the linear regression models to predict MCS. The three independent variables were entered using forward stepwise regression (see model 1–3). The analysis in model 2 showed an inverse association with psychological distress (SCL-K-9) and a positive association with self-esteem (RSES). Furthermore, the analysis in model 3 revealed that stressful life events (SRRS) did not provide significant additional information to predict MCS. The explained variance of the model was 78% (R-squared = 0.78).

**Table 5:** Regression models for MCS.

	*Beta*	*SD*	*t*-value
Model 1: MCS ~ SCL			
SCL	−1.370	0.143	−9.617 ^***^
Model 2: MCS ~ SCL + RSES			
SCL	−1.152	0.182	−6.344 ^***^
RSES	0.421	0.231	1.824 ^*^
Model 3: MCS ~ SCL + RSES + SRRS			
SCL	−1.184	0.216	−5.490 ^***^
RSES	0.403	0.243	1.658
SRRS	0.002	0.001	0.281

The analysis of all variables was conducted with linear regression models. MSC: Mental Health Score of SF-36: Short Form 36; SCL-K-9: Symptom Checklist short version-9; SRRS: Social Readjustment Rating Scale; RSES: Rosenberg Self Esteem Scale.

* indicates *p* < 0.1, ** indicates *p* < 0.01, *** indicates *p* < 0.001.

## Discussion

To our knowledge, this is the first study to examine the quality of life (QoL) (aim 1) and simultaneously the mental health status, psychological stress level and stressful situations (aim 2) in Swiss trans people during their transition. A special focus was given on the impact of self-esteem and life events’ impact on QoL (aim 3). As assumed, trans individuals showed below average mental health QoL levels. The results indicate that trans individuals are exposed to a high degree of psychological burden and that a large amount of trans persons have poor mental health with a large spectrum of variability and a high prevalence of psychiatric diagnoses. Furthermore, participants reported more stress, especially in social (job loss and moving in a new home) and psycho-somatic domains (change of eating and sleeping habits). Finally, the results suggest significant relationships between life events, self-esteem, psychological distress and QoL in transitioning individuals which is in concordance with the gender minority stress model (GMSM).

### Low QoL during transition

Corresponding to an earlier study on Swiss trans persons, participants had a lower QoL compared to the cis population (16). However, while mental health-related QoL (MCS) of the trans feminine groups in both studies hardly differed from each other, our trans masculine group showed lower MCS values. This difference could be related to the differing participants’ transition stages in both surveys. While this study included trans individuals who were in social and/or medical transition, Jellestad et al. examined a cohort of post-transitioned trans persons (16).

A comparison of our results with the QoL of people with serious medical condition shows that patients with very severe chronic obstructive pulmonary disease have a similarly poor mental health-related QoL (MCS: 47.7 ± 4.4) (39). Similarly, individuals suffering from multiple sclerosis without fatigue show a QoL (MCS: 46.6) on the same level as our cohort (40). In contrast, chronic pain patients, individuals suffering from schizophrenia and persons with obsessive-compulsive disorders have a lower MCS (M ± SD) (41–43). The interpretation of these data is not straightforward, because although many of the people studied had one or more psychiatric disorders, they were already undergoing transitional medical treatment, which can have a positive impact on both mental health and QoL. Nevertheless, the persistence of deficits in QoL suggests that the entire transition process may still pose a significant burden.

### Multiple and diverse mental health issues and life stressors

The study population shows a high prevalence of depressive and especially anxiety disorders as well as self-damaging behaviors such as attempted suicide. These results correspond to the observations from foreign studies (44) and reflect the data from other research in Switzerland (45–48) where up to 45.2% of the participants suffered from mood disorders and up to 16.7% had previously had suicidal thoughts. Especially the amount of people (mostly trans feminine: 60%) affected by anxiety disorders (in particular, social phobia and generalized anxiety disorders) is alarming.

In our view, this high rate and type of anxiety disorders could be rather a result of the exposure to transition-related stigma than of a higher predisposition to anxiety disorders of the trans population. Due to the changes regarding appearance and perception during transition, trans individuals may be exposed to (more) stigmatization and may experience distress when staying in public space (19). As a result, they can internalize these messages in the sense of a “felt stigma” (48) and develop expectations about their own situation and consequently social avoidance behaviors. If one considers these symptoms in a decontextualized manner, as the MINI-PLUS does, then there is a risk that these understandable psychological defense reactions will be viewed as autonomous psychopathology. Consequently, a stigma aware psycho-diagnostic instrument would be needed to get more evidence regarding social phobia and generalized anxiety disorders for trans individuals.

Most of the stressors were recorded in the social sphere: trans persons are especially confronted with financial and housing problems and couple conflicts, which again shows that in some cases the transition sets off a complex dynamic of trans-negative exclusion that can lead to social withdrawal. Trans individuals with lower socioeconomic status may also more affected by housing problems and job loss than those with high socioeconomic status due to their lower financial reserves (15, 17–19). In addition, this effect may be amplified by the below average trans-protective laws in Switzerland compared to other OECD countries (49). A qualitative study carried out in parallel supports this supposition (50). Trans individuals reported that many of the events mentioned above were related to their social transition and that the consequences of certain life events are also reflected in the high rates of stress related behavioral changes (e. g. sleep and eating disturbances). Even if the life events’ impact on participants was at most moderate (for trans people recruited in the hospital), it is comparable to that of patients who suffer from serious health problems such as ankylosing spondylitis (51) or schizophrenia (52).

### Is there a connection between stressors and QoL?

A positive association was found between self-esteem and QoL (corresponding with the negative correlation between RSES and MCS due to the reversed scale of the RSES). As expected, the picture was reversed regarding the reported stress experiences and psychological distress: they were both negatively associated with QoL. These dynamics are also seen in other medical conditions, where elevated stress levels correlate with a poor QoL (53). There are also studies that show a negative correlation between distal stressors (e.g., stigmatization), proximal stressors (e.g., self-stigmatization) and mental health in trans persons (46). In this study, stressful life events and self-esteem are surrogates of distal and proximal stressors, respectively. Although these surrogates are not trans-specific, the circumstance that they were applied during the transition phase turns them to our opinion into feasible instruments to measure these stressors. Accordingly, our findings provide another indication of the validity of the GMSM.

In concordance with the correlation analyses, the regression analysis revealed that psychological distress and self-esteem are significant predictors of QoL, however, the stressful life events (SRRS) seem not due to a too weak statistical power. Furthermore, the regression analysis could confirm the interaction between the proximal stressors and QoL in view of the GMSM, but statistical power was also here too weak for the distal ones.

## Limitations

Some limitations must be addressed. First, the small number of participants – even if greater than in past studies in Switzerland (45, 47) – limits the generalization of our results. Second, recruitment bias could have skewed the results as participants in hospitals tend to have more mental health problems and a lower QoL compared to the general trans population. This risk had been reduced by including participants outside the university clinic. However, the results showed no statistically significant difference between these groups. Third, in the absence of an instrument validated in German that could have measured the gender minority stress burden, a well-known instrument in stress research was chosen to directly measure the impact of distal live events. Although the participants associated many of the life events with their social transition, the utilization of the SRRS caused our data to also include a broad spectrum of negative situations and not only those generally faced by gender minorities. Therefore, a specialized tool with more selective parameters should be used in future studies. Finally, the cross-sectional nature of the study does not allow us to draw conclusions on the etiology of the phenomenon. Accordingly, a larger, prospective study would be needed to deepen our knowledge on the Swiss trans population. Thus, further research addressing this important question of the actual extent of the psychiatric burden on trans individuals is required.

## Conclusion

Our results have shown a low QoL for trans individuals associated with low self-esteem and stressful life events. These demand that the transition process should not be considered a solely “medical” matter, but a multifactorial one including psychological, physical and social processes. Therefore, more systemic interventions (e.g., involvement of family members, school, employers) provided by the care system but also by peers are needed.

Moreover, our findings indicate a disproportionately high rate of anxiety disorders and depression in the cohort studied and point to the important role of self-esteem, stigmatization by self and others, and the impact that nonconformity with cis heteronormative norms can have. For these reasons, therapists should not focus on isolated gender incongruence symptoms. In contact with trans people, it is important to integrate the impact of the underlying stigma to which they are permanently exposed (54).

## Data availability statement

The raw data supporting the conclusions of this article will be made available by the authors, without undue reservation.

## Ethics statement

The studies involving humans were approved by Kantonale Ethikkommission Zürich. The studies were conducted in accordance with the local legislation and institutional requirements. The participants provided their written informed consent to participate in this study.

## Author contributions

GF: data analysis and writing. LP: data analysis, writing, interpretation, and editing. TJ: supervision of data analysis. VS: supervision of the manuscript. RvK: supervision of the manuscript. DGN: conceptualization, interpretation supervision, and writing of the manuscript. All authors contributed to the article and approved the submitted version.

## Footnotes

^1^A person whose sense of personal identity and gender corresponds with their assigned sex at birth.
